# Supplementary material for: Content-rich biological network constructed by mining PubMed abstracts
Source: BMC Bioinformatics. 2004 Oct 8;5:147. doi: 10.1186/1471-2105-5-147 (PMC528731; doi:10.1186/1471-2105-5-147)
Supplement: Additional File 2 — The original results of the above study (non-essential files are deleted to keep the file size under the limit set by BMC bioinformatics). [file 1471-2105-5-147-S2.bz2 › chilibotAdditionalFile2/dip05/385ID9252374E9850/html/THPO_MPL.html]

 


 **THPO** and **MPL** 
  
Found 539 abstracts in PubMed, retrieved 05.  
 

 What does Google say? 
 PDF only 
| .edu only 

---

**Interactive relationship** (e.g. stimulation, inhibition, etc)

**Inhibitory relationship**- Our case report suggests that AMT in patients with SSc could be mediated by the anti c  **Mpl**   [ **MPL** ]  antibody, which functionally blocks an interaction between thrombopoietin  [ **THPO** ]  and c  **Mpl**   [ **MPL** ] .  Ref: 12794833 Arthritis Rheum, 2003
**Stimulatory relationship**- Although it is clear that thrombopoietin  [ **THPO** ]  is the primary regulator of thrombopoiesis, several lines of evidence indicate that the hormone affects multiple aspects of hematopoiesis the in vivo administration of TPO  [ **THPO** ]  increases marrow levels of erythroid, myeloid, and megakaryocytic progenitor cells and its genetic elimination or that of its receptor c  **mpl**   [ **MPL** ]  reduces the numbers of these cells.  Ref: 12799280 Ann N Y Acad Sci< MedlineTA>Ann N Y Acad Sci, 2003
**Neutral relationship**- Thrombopoietin  [ **THPO** ]  TPO  [ **THPO** ]  is a hematopoietic cytokine that regulates megakaryocytosis and thrombocytosis by binding to its receptor c  **Mpl**   [ **MPL** ] .  Ref: 12841634 Mol Cell Biochem, 2003

**Non-interactive relationship** (e.g. studied together, co-existance, homology, etc.)

- Like CAMT patients, mice lacking the TPO  [ **THPO** ]  receptor c  **Mpl**   [ **MPL** ]  demonstrate a major reduction of early hematopoietic progenitor cells of all lineages.  Ref: 12799278 Ann N Y Acad Sci, 2003
- Anti c  **Mpl**   [ **MPL** ]  thrombopoietin  [ **THPO** ]  receptor autoantibody induced amegakaryocytic thrombocytopenia in a patient with systemic sclerosis.  Ref: 12794833 Arthritis Rheum, 2003
- Her serum was strongly positive for anti c  **Mpl**   [ **MPL** ]  antibody, and IgG fraction purified from her serum inhibited thrombopoietin  [ **THPO** ]  dependent cell proliferation in vitro.  Ref: 12794833 Arthritis Rheum, 2003
- Because thrombopoietin  [ **THPO** ]  TPO  [ **THPO** ]  and its receptor, c  **Mpl**   [ **MPL** ] , regulate primitive hematopoietic populations, including bone marrow hematopoietic stem cells, we investigated whether TPO  [ **THPO** ]  acts on the hemangioblasts that derive from differentiation of embryonic stem cells in vitro.  Ref: 12743322 Stem Cells, 2003
- Here we describe a rare case of SSc accompanied by thrombocytopenia and megakaryocytic hypoplasia, in which autoantibody against thrombopoietin  [ **THPO** ]  receptor c  **Mpl**   [ **MPL** ]  was detected.  Ref: 12794833 Arthritis Rheum, 2003
